# Parental Diseases of Despair and Suicidal Events in Their Children

**DOI:** 10.1001/jamanetworkopen.2025.31442

**Published:** 2025-09-12

**Authors:** David A. Brent, Kwan Hur, Jason B. Gibbons, Giovanna Porta, Robert D. Gibbons

**Affiliations:** 1 Department of Psychiatry, University of Pittsburgh School of Medicine, Pittsburgh, Pennsylvania; 2 Center for Health Statistics, University of Chicago, Chicago, Illinois; 3 Division of Pharmacoepidemiology and Pharmacoeconomics, Brigham and Women’s Hospital, Harvard Medical School, Boston, Massachusetts; 4 University of Pittsburgh Medical Center, Western Psychiatric Hospital, Pittsburgh, Pennsylvania

## Abstract

**Importance:**

Suicide and suicidal behavior among US adolescents has increased dramatically over the past 2 decades, without a clear explanation. Deaths of despair in midlife adults, due to suicide, alcohol-related disease, or drug overdose, have doubled in the past decade, with parallel increases in corresponding diseases of despair (DoD), indicating that there could be an association between these 2 epidemics.

**Objective:**

To assess associations of parental DoD with suicidal events (SE) in their offspring.

**Design, Setting, and Participants:**

This retrospective cohort study used the MarketScan commercial claims and encounter database, encompassing 164 million individuals, with claims data from 2010 to 2020. Prelinked parents aged 30 to 50 years and their children aged 8 to 15 years were included. Data were analyzed from November 2023 to May 2025.

**Exposure:**

Parent with or without a DoD, defined as a suicide attempt, alcohol-related disease, or substance use disorder according to diagnostic codes.

**Main Outcomes and Measures:**

The primary outcome was child SE, either a suicide attempt or self-harm, adjusted for age and sex. Children were followed until their SE, disenrollment from commercial health insurance, or 730 days after the parental DoD index diagnosis. Data were analyzed using a Cox proportional hazards model, with families with and without DoD balanced in terms of potential confounders through inverse probability weighting.

**Results:**

The study included 561 837 families with at least 1 parent diagnosed with a DoD (291 463 male [51.9%]; 244 943 [43.6%] aged 30-39 years) and 1 180 546 control families (591 976 male [50.1%]; 498 778 [42.2%] aged 30-39 years), from which 817 133 children from families with DoD (417 770 male [51.1%]; 383 810 [47.0%] aged 8 to 11 years) and 1 744 182 children from control families (889 308 male [51.0%]; 884 749 [50.7%] aged 8-11 years) were identified. Exposure to parental DoD was associated with an increased hazard for an SE (hazard ratio [HR], 1.67; 95% CI, 1.54-1.82). Youths with 2 parents with DoD had a larger hazard for an SE than those with 1 affected parent (interaction HR, 1.95, 95% CI, 1.58-2.39). There was a significant age by sex interaction in girls aged 8 to 11 years (HR, 3.12; 95% CI, 2.05-4.74) but not boys (HR, 0.99; 95% CI, 0.63-1.54). Maternal DoD was associated with a higher risk for a child SE than paternal DoD (interaction HR, 1.44; 95% CI, 1.13-1.84).

**Conclusions and Relevance:**

This cohort study found an association of parental DoD with youth SE; this finding may be underlying the increase in adolescent suicidal behavior observed in the US over the past 2 decades. Improved access to care for parents with DoD and systematic screening and referral of their offspring could help to reduce the adolescent suicide rate.

## Key Points

**Question:**

Are parental diseases of despair (DoD; ie, substance use disorder, alcohol-related disease, or suicidal behavior) associated with an increased risk for suicidal events in their children?

**Findings:**

In this cohort study of 561 837 parents with DoD and 1 180 846 parents without DoD, coming from a family with a parent with DoD was associated with an increased hazard of suicidal events in exposed children.

**Meaning:**

The findings of this study suggest that the rise in teenage suicide and suicidal behavior may be in part associated with concomitant increases in parental DoDs.

## Teaser

This cohort study examines associations of substance use disorder, alcohol-related disease, or suicidal behavior (ie, diseases of despair) among parents with suicidal events in their children.

## Introduction

The suicide rate among US adolescents has increased by 60% from 2007 to 2020,^1^ with parallel increases in suicidal ideation and behavior.^2,3^ There has been a particularly sharp increase in suicide in preteenage and early adolescent racially and ethnically minoritized youths and in girls.^4,5,6^ The causes of these concerning trends are unknown, making it difficult to develop prevention strategies.

The rise in adolescent suicide could be associated with another epidemic affecting midlife adults, namely, deaths of despair, consisting of deaths due to drug overdoses, alcohol-related disease, and suicide. Case and Deaton^7^ first introduced the term *deaths of despair* based on their observation that, in contrast with other wealthy countries, the US was showing a decline in life expectancy, driven by an increase in mortality in midlife adults due to the aforementioned causes. The mortality rate due to deaths of despair has more than doubled in those aged 45 to 54 years from 1998 to 2022.^8^ While initially, deaths of despair were most often found among White males without a college education, this epidemic now affects females as well as males, and has spread across Black, Hispanic, American Indian and Alaska Native, and less socially vulnerable White populations.^8,9^ In the past decade, more than 300 000 children have lost their parents due to drug overdoses alone.^10^ For every death of despair, there are many more adults affected by diseases of despair (DoD), namely alcohol use disorder and alcohol-related diseases (ARDs), substance use disorders (SUDs), and suicide attempts (SAs). One study^11^ analyzed trends in insurance claims from 2009 to 2018, finding that among adults of parental age (35-54 years), SUDs increased by more than 50%, while SAs increased by 81% in males and 151% in females.

Few studies have examined the association of parental DoD with adolescent suicide. Powell^12^ showed that the state-level frequency of pre-2010 oxycontin misuse was associated with as much as 49% of the increase in adolescent suicide in that state over the next decade. These results, while compelling, do not directly associate parental opiate misuse with adolescent suicide.

This association of parental DoD with adolescent suicidal behavior is plausible, because the of parents with a history of SA, alcohol use disorder, or opiate misuse have approximately twice the rate of SA as children without exposure to parental DoD.^13,14,15^ Possible mechanisms include the familial transmission of comorbid disorders and liability to suicidal behavior from parents with DoD to their children, thereby increasing their children’s risk for suicidal behavior.^16,17,18,19^ Children exposed to parental DoD are more likely to experience neglect, abuse, exposure to interpersonal violence, parental incarceration, removal from their home, and parental death, all of which are significant risk factors for suicidal behavior.^20,21,22,23,24^

In this study, we examined the association of parental DoD with suicidal events (SEs; ie, SA or self-harm) in their children using a large insurance claims database that linked parent and child claims. We hypothesized that the children of parents with ARD, SUD, and/or SA would show a 2-fold increase in SE, thus supporting an association of the increases in parental DoD with in adolescent suicidal behavior.

## Methods

### Study Design

This cohort study was determined exempt by the University of Chicago institutional review board due to the use of deidentified data. Reporting followed the Strengthening the Reporting of Observational Studies in Epidemiology (STROBE) reporting guideline. We analyzed MarketScan Commercial Claims and Encounters databases,^25^ distributed by Merative, which include inpatient, outpatient, and prescription claims from more than 100 US insurers (164 million unique enrollees from 2005 to 2017). Diagnostic codes for DoD (ie, SA and intentional self-harm, SUD, and ARD) and potential confounders of concomitant neuropsychiatric disorders were based on *International Classification of Diseases, Ninth Revision, Clinical Modification (ICD-9-CM)* and *International Statistical Classification of Diseases, Tenth Revision, Clinical Modification (ICD-10-CM)*. A list of *ICD-9-CM* and *ICD-10-CM* codes utilized to identify DoD is provided in eTable 1 in Supplement 1. The data used in these analyses included 2011 to 2020, but data were pulled from 2010 to 2021 so that each patient could have at least 1 year of historical data and 1 year of follow-up.

We identified 807 261 families with at least 1 parent with DoD, and 6.82 million families without DoD. Parents were aged 30 to 50 years. All families had 1 or more children. 61 408 families had 2 adults with DoD (7.6%), of whom we randomly selected 1 adult per family to utilize in the family matching. We randomly selected up to 3 families without DoD, and 1 adult within these families, based on the index year of the DoD diagnosis, sex and age of the selected parent, and location of the family. We estimated logistic regression models to generate propensity scores for the probability of a child belonging to a DoD family as a function of pre-DoD parental diagnoses and psychotropic medication use. Thereafter, we conducted survival analyses with and without inverse probability weighting (IPW) to compare time to the first SE in offspring as a function of exposure to parental DoD. There were 245 424 cases that were lost due to inability to match on location. Information on race and ethnicity was not available.

Frequencies of the potential demographic, disease condition, and medication confounders in families with and without DoD before and following weighting were calculated. Before applying IPW, there were no demographic differences, but there were large differences in the incidence of neuropsychiatric disorders and related medication use. Following IPW, the groups were well-balanced.

From these families, we identified children aged 8 to 15 years of parents with and with and without DoD. We chose 8 years as the lower limit considering reports of increases in preadolescent suicide,^6^ and an upper age of 15 years to allow for up to 2 years of follow-up while children were still living at home.

**Figure 1.**
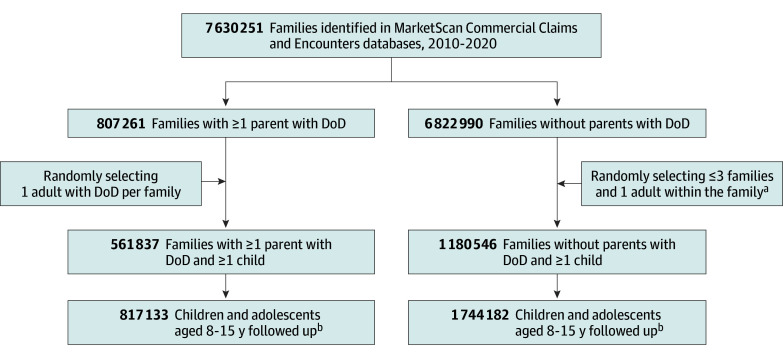
Participant Flow ^a^Based on the index year of the family with diseases of despair (DoD), sex, age, and location. ^b^Until the first suicidal event, disenrollment from commercial insurance, or 730 days after the parental DoD index diagnosis.

Figure 1 shows the flow of participants into the study. Age and sex, rates of psychiatric diagnoses, and psychotropic medication use for children from families with and without DoD with and without weighting were calculated.

### Outcomes

Our primary outcome was an SE in the child resulting in an outpatient visit or inpatient admission as identified by the *ICD-9-CM* or *ICD-10-CM* codes listed in eTable 1 in Supplement 1. Median (IQR) follow-up times for participants with and without DoD were 496 ( 244-730) and 493 (247-730) days, respectively. The primary outcome analysis was both weighted and adjusted for child age and sex.

### Exposures

The primary exposure was having at least 1 parent who was diagnosed with a DoD. Youths who had parents with DoD were followed from the parental index DoD diagnosis to the child’s first SE, disenrollment from commercial insurance, or 730 days. Similarly, children whose parents did not have DoD were followed from the index diagnosis of the matched parent with to their first SE, commercial insurance disenrollment, or 730 days.

### Statistical Analysis

Statistical analysis was conducted from November 2023 to May 2025 using SAS 9.4 (SAS Institute). Statistical significance was defined as a 2-sided *P* < .05.

#### Main Analysis

The data were analyzed using a Cox proportional hazards model for time to the first SE. We utilized a total of 6 different model specifications across 3 age groups (8-15, 8-11, and 12-15 years) for a total of 18 different models. The first 3 model specifications excluded IPW ^26^ and consisted of an unadjusted (for child variables) model, a model adjusted for child age and sex, and a model adjusted for child age, sex, and baseline child disease conditions. Baseline child disease conditions included DoD (ARD, SUD, and SA), depression, anxiety disorder, attention-deficit/hyperactivity disorder, schizophrenia, and bipolar disorder.

We then reestimated each of these 3 model specifications after incorporating IPW using the parent baseline characteristics. The weights were computed by logistic regression (ie, inverse of a propensity score) and were stabilized by dividing the weights by the mean weight and truncating them at the 0.1 and 99.9 percentiles. Because many of the potential confounders are correlated with DoD and increase the risk for child SE (eg, depression), we posited that the unweighted and weighted analyses were approximate upper and lower bounds on the association of parental DoD with child SE. Only 9 families had more than 1 SE, obviating the need to adjust for familial clustering. As a sensitivity analysis we used a generalized estimating equation model to confirm the results of the primary analysis with and without clustering.

#### Demographic Factors and Moderators

To examine the relative impact of having 1 vs 2 parents with DoD, we stratified the sample accordingly and conducted Cox proportional hazards models both weighted and unweighted, unadjusted, adjusted for child age and sex, and adjusted for child age, sex, and psychopathology. To explore whether child sex and age moderated the association of DoD with suicidal events, we conducted an additional analysis that included interactions among sex, age, and DoD exposure. To examine whether there was a differential effect of exposure to maternal or paternal DoD, we compared hazard ratios (HRs) for SEs for youths exposed to maternal vs paternal DoD in families with 1 parent with DoD.

## Results

Random selection resulted in 561 837 parents with DoD and 1 180 846 parents without DoD. In the unweighted sample, among parents with DoD, 291 463 (51.9%) were male and 244 943 (43.6%) were aged 30 to 39 years, while among parents without DoD, 591 976 (50.1%) were male and 498 778 (42.2%) were aged 30 to 39 years (Table 1). The distribution of DoDs among parents included 3 612 265 parents (64.3%) with SUD, 185 484 parents (33.0%) with ARD, 8839 parents (1.6%) with both SUD and ARD, and 6149 parents (1.1%) with an SA with or without comorbid ARD or SUD. From these families, 817 133 children of parents with DoD (751 510 and 65 623 children exposed to 1 and 2 parents with DoD, respectively) and 1 744 182 children of parents without DoD were identified. In the unweighted sample, among offspring of parents with DoD, 417 770 (51.1%) were male and 383 810 (47.0%) were aged 8 to 11 years; while among offspring of parents without DoD, 889 308 (51.0%) were male and 884 749 (50.7%) were aged 8 to 11 years (Table 2). Families with DoD had proportionally more children aged 12 to 15 years (433 323 children [53.0%] vs 859 433 children [49.3%]). Rates of neuropsychiatric disorders and of prescribed psychotropic medications were all significantly higher in children of parents with DoD children both before and after weighting (Table 2).

**Table 1.** Adults’ Baseline Characteristics Before and After Weighting

Variable	Adults, No. (%) [Unweighted]		Standardized difference (%)	Adults, No. (%) [Weighted]		Standardized difference (%)
	Non-DoD (n = 1 180 546)	DoD (n = 561 837)		Non-DoD (n = 857 725)	DoD (n = 847 671)	
Age, y						
30-39	498 778 (42.2)	244 943 (43.6)	2.72	370 408 (43.2)	367 675 (43.4)	0.38
40-39	681 768 (57.8)	316 894 (56.4)	2.72	487 317 (56.8)	479 995 (56.6)	0.38
Sex						
Male	591 976 (50.1)	291 463 (51.9)	3.47	437 785 (51.0)	435 299 (51.4)	0.62
Female	588 570 (49.9)	270 374 (48.1)	3.47	419 941 (49.0)	412 371 (46.6)	0.62
Disease conditions						
Depression	37 653 (3.2)	105 672 (18.8)	51.55	73 501 (8.6)	71 431 (8.4)	0.51
Anxiety disorder	56 635 (4.8)	128 906 (22.9)	54.41	93 625 (10.9)	92 227 (10.9)	0.11
ADHD	9594 (0.8)	17 582 (3.1)	16.72	13 831 (1.6)	13 727 (1.6)	0.05
Schizophrenia	210 (0)	1080 (0.2)	5.39	571 (0.1)	640 (0.1)	0.34
Bipolar disorder	5460 (0.5)	24 875 (4.4)	25.89	14 073 (1.6)	15 064 (1.8)	1.05
Medications						
Hypnotic^a^	15 400 (1.3)	29 450 (5.2)	22.26	23 109 (2.7)	22 715 (2.7)	0.09
ADHD	10 563 (0.9)	17 830 (3.2)	16.20	14 346 (1.7)	14 348 (1.7)	0.16
Antidepressants	75 201 (6.4)	131 533 (23.4)	49.3	196 448 (12.4)	104 533 (12.3)	0.24
Antipsychotics	3653 (0.3)	18 945 (3.4)	22.93	10 521 (1.2)	11 420 (1.3)	1.07
Mood stabilizer	2903 (0.2)	9609 (1.7)	14.92	5657 (0.7)	6345 (0.7)	1.06
Gabapentinoid	5516 (0.5)	26 877 (4.8)	27.25	14 267 (1.7)	15 997 (1.9)	1.69
Nonbenzodiazepine anxiolytic	6546 (0.6)	19 962 (3.6)	21.26	12 669 (1.5)	13 259 (1.6)	0.71
Antiepileptic	8062 (0.7)	18 372 (3.3)	18.67	12 852 (1.5)	13 266 (1.6)	0.54
Muscle relaxant	34 996 (3)	74 285 (13.2)	38.29	53 376 (6.2)	53 967 (6.4)	0.59
Tobacco cessation	2041 (0.2)	19 819 (3.5)	25.09	9810 (1.1)	10 754 (1.3)	1.15
Benzodiazepine	15 157 (1.3)	45 932 (8.2)	32.90	31 517 (3.7)	30 938 (3.6)	0.13

Abbreviation: ADHD, attention-deficit/hyperactivity disorder.

^a^ Zolpidem, zaleplon, and eczopiclone.

**Table 2.** Children’s Baseline Characteristics Without and With Weighting

Variable	Children, No. (%) [Unweighted]			Children, No. (%) [Weighted]			Odds of having a parent with DoD, OR (95% CI)
	Non-DoD (n = 1 744 182)	DoD (n = 817 133)	*P* value	Non-DoD (n = 1 266 755)	DoD (n = 1 229 471)	*P* value	
Age, y							
8-11	884 749 (50.7)	383 810 (47.0)	NA	644 129 (50.9)	574 177 (46.7)	NA	1 [Reference]
12-15	859 433 (49.3)	433 323 (53.0)	<.001	622 625 (49.2)	655 294 (53.3)	<.001	1.18 (1.18-1.19)
Sex							
Male	889 308 (51.0)	417 770 (51.1)	.04	646 546 (51.0)	627 808 (51.1)	.71	1.00 (1.00-1.01)
Female	854 874 (49.0)	399 363 (48.9)		620 909 (49.0)	601 663 (48.9)		1 [Reference]
Disease conditions							
Disease of despair	1334 (0.1)	2600 (0.32)	<.001	1119 (0.09)	3495 (0.28)	<.001	3.21 (3.01-3.44)
Depression	11 125 (0.6)	15 264 (1.9)	<.001	10 088 (0.8)	17 773 (1.5)	<.001	1.83 (1.78-1.87)
Anxiety disorder	40 270 (2.3)	44 999 (5.5)	<.001	35 028 (2.8)	52 927 (4.3)	<.001	1.58 (1.56-1.60)
ADHD	64 111 (3.7)	65 456 (8.0)	<.001	53 978 (4.3)	84 372 (6.9)	<.001	1.66 (1.64-1.67)
Schizophrenia	68 (<0.0)	148 (<0.0)	<.001	63 (<0.0)	176 (<0.0)	<.001	2.89 (2.17-3.86)
Bipolar disorder	5640 (0.3)	8460 (1.0)	<.001	5405 (0.4)	9833 (0.8)	<.001	1.88 (1.82-1.95)
Medications							
Hypnotic^a^	100 (0.01)	178 (<0.0)	<.001	101 (<0.0)	197 (<0.0)	<.001	2.00 (1.57-2.54)
ADHD	60 823 (3.5)	52 624 (6.4)	<.001	52 190 (4.1)	63 729 (5.2)	<.001	1.27 (1.26-1.29)
Antidepressants	16 527 (1.0)	17 497 (2.1)	<.001	15 471 (1.2)	18 826 (1.5)	<.001	1.26 (1.23-1.28)
Antipsychotics	6739 (0.4)	7840 (1.0)	<.001	6497 (0.5)	8646 (0.7)	<.001	1.37 (1.33-1.42)
Mood stabilizer	1760 (0.1)	1876 (0.2)	<.001	1652 (0.1)	2091 (0.2)	.003	1.30 (1.22-1.39)
Gabapentinoid	516 (<0.0)	531 (0.1)	<.001	482 (<0.0)	601 (0.1)	<.001	1.28 (1.14-1.45)
Nonbenzodiazepine anxiolytic	3503 (0.2)	4304 (0.5)	<.001	2963 (0.2)	4999 (0.4)	<.001	1.74 (1.66-1.82)
Antiepileptic	6135 (0.4)	5360 (0.7)	<.001	5160 (0.4)	6371 (0.5)	<.001	1.27 (1.23-1.32)
Muscle relaxant	1853 (0.1)	2158 (0.3)	<.001	1547 (0.1)	2624 (0.2)	<.001	1.75 (1.64-1.86)
Tobacco cessation	0	5 (0.00)	.003	0	4 (<0.0)	.03	NA
Benzodiazepine	1640 (0.1)	1732 (0.2)	<.001	1441 (0.1)	1882 (0.2)	<.001	1.34 (1.26-1.44)

Abbreviations: ADHD, attention-deficit/hyperactivity disorder; OR, odds ratio; NA, not applicable.

^a^ Zolpidem, zaleplon, and eczopiclone.

**Figure 2.**
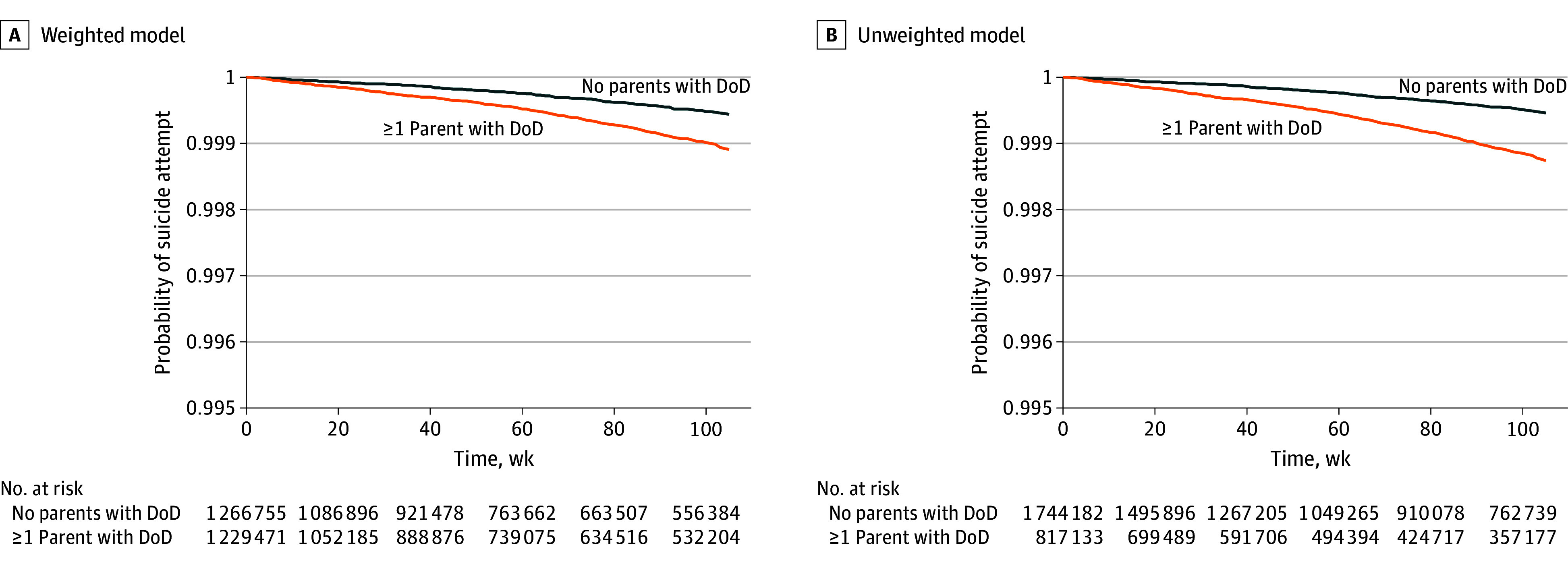
Probability of Suicide Attempt by Group DoD indicates diseases of despair.

Table 3 shows the distribution of SEs among youths of parents with and without DoD and presents HRs for unweighted and weighted analyses, unadjusted, adjusted for child age and sex, and adjusted for child age, sex, and psychopathology. Our primary analysis, weighted and adjusted for child age and sex, indicated an increased hazard for time to first SE of (HR, 1.67; 95% CI, 1.54-1.82), with the corresponding Kaplan-Meier curve in Figure 2. We posited that the upper and lower bounds of the association of parental DoD with time to child SE would be represented by estimates that were unweighted and unadjusted (HR, 2.26; 95% CI, 2.08-2.45) and weighted and adjusted for child age, sex, and psychopathology (HR, 1.48; 95% CI, 1.36-1.61), respectively. Similar results were obtained when analyzed separately for ages 8 to 11 years and ages 12 to 15 years (Table 3). The generalized estimating equation analysis showed no differences between models that did and did not incorporate familial clustering, and those estimates were virtually identical to the primary analytic results (eTable 2 in Supplement 1).

**Table 3.** Rate and Hazard of Suicide Event Among Children of Parents With and Without DoD

Age group by weighting and category	Children, No.	Events, No.	Days, No.	Rate per 10 000 person-years	Hazard of suicidal event		
					Model 1, unadjusted HR (95% CI)	Model 2, adjusted HR (95% CI)^a^	Model 3, adjusted HR (95% CI)^b^
All ages							
Unweighted							
Non-DoD	1 744 182	1145	850 864 291	4.91	1 [Reference]	1 [Reference]	1 [Reference]
DoD	817 133	1210	398 302 891	11.09	2.26 (2.08-2.45)	2.10 (1.93-2.27)	1.68 (1.54-1.82)
Weighted							
Non-DoD	1 266 755	888	619 071 408	5.23	1 [Reference]	1 [Reference]	1 [Reference]
DoD	1 229 471	1555	597 181 621	9.51	1.82 (1.68-1.98)	1.67 (1.54-1.82)	1.48 (1.36-1.61)
Ages 8-11 y							
Unweighted							
Non-DoD	884 749	82	430 347 085	0.70	1 [Reference]	1 [Reference]	1 [Reference]
DoD	383 810	97	186 699 201	1.90	2.73 (2.03-3.66)	2.64 (1.97-3.55)	2.22 (1.64-3.00)
Weighted							
Non-DoD	644 129	72	313 744 187	0.83	1 [Reference]	1 [Reference]	1 [Reference]
DoD	574 177	119	278 361 572	1.56	1.88 (1.40-2.52)	1.82 (1.36-2.44)	1.65 (1.23-2.22)
Ages 12-15 y							
Unweighted							
Non-DoD	859 433	1063	420 517 206	9.23	1 [Reference]	1 [Reference]	1 [Reference]
DoD	433 323	1113	211 603 690	19.20	2.08 (1.91-2.26)	2.06 (1.89-2.24)	1.64 (1.50-1.78)
Weighted							
Non-DoD	622 625	816	305 327 221	9.76	1 [Reference]	1 [Reference]	1 [Reference]
DoD	655 294	1436	318 820 049	16.44	1.69 (1.55-1.84)	1.66 (1.53-1.81)	1.47 (1.35-1.60)

Abbreviations: DoD, diseases of despair; HR, hazard ratio.

^a^ Adjustment for age and sex.

^b^ Adjustment for age, sex, and baseline disease conditions.

Youths exposed to 2 parents with DoD had approximately twice HR of an SE compared with youths with 1 parent with DoD, although both groups of DoD-exposed youths had an increased hazard (interaction HR, 1.95; 95% CI, 1.58-2.39) (eTable 3 in Supplement 1). For example, in the most conservative analysis, weighted and adjusted for child age, sex, and psychopathology, children exposed to 1 parent with DoD had an 18% increased hazard (HR, 1.18; 95% CI, 1.06-1.31) and children exposed to 2 parents with DoD had a 106% increased hazard (HR, 2.06; 95% CI, 1.72-2.46).

The HRs stratified by sex and age are displayed in eTable 4 in Supplement 1. There was a statistically significant interaction between DoD, age, and sex interaction (HR, 3.11; 95% CI, 2.05-4.73). Specifically, there was a significant interaction of parental DoD for females aged 8 to 11 years but not for similarly aged males (weighted, unadjusted HR, 3.12; 95% CI, 2.05-4.74 vs HR, 0.99; 95% CI, 0.63-1.54). Exposure to maternal vs paternal DoD (weighted, adjusted for child’s age and sex) was associated with a significantly increased hazard for an SE (interaction HR, 1.44; 95% CI, 1.13-1.84).

## Discussion

In this cohort study of parent and child–linked claims data, we found that having a parent with a DoD was associated with nearly double the hazard of an SE in their offspring. This finding supports our hypothesis that the epidemics of DoD in midlife adults and suicidal behavior in youths are associated. This association was robust, even after matching on parental psychopathology and adjusting for child age, sex, and psychopathology, suggesting that the environmental factor of parental DoD plays a significant role in child SEs. Having 2 parents with DoD was associated with approximately twice the HR for an SE compared with those with single parent DoD exposure. The association of parental DoD with SE was present in girls aged 8 to 11 years, but there was no association for same-aged . Exposure to maternal DoD conveyed a greater risk for SEs than did exposure to paternal DoD.

While this study cannot establish a causal relationship between parental DoD and youth SE, there are plausible pathways from parental DoD to offspring suicidal behavior, namely transmission of comorbid psychiatric disorder ^16,17,18^ and liability to suicidal behavior,^16,19^ exposure to adverse childhood experiences associated with parental DoD,^20,21^ and increased risk for parental death,^7,8,10^ all of which can raise the risk of suicidal behavior in offspring.^13,14,15,16,17,18,19,20,21,22,23,24^

Although not hypothesized a priori, the association of parental DoD with SEs in girls aged 8 to 11 years-o is consistent with national trends showing that girls aged 10 to 14 years are experiencing the most rapid increases in suicide in young people.^6^ There is evidence, not necessarily sex-specific, that parent loss and psychopathology are more deleterious in younger children.^13,16,22,23^ The finding that maternal DoD appears to have a greater adverse impact on children than paternal DoD is consistent with several other studies showing a greater negative impact of maternal vs paternal loss or impairment on youth mental health.^13,18,19,22^

Exposure to parental DoD cannot completely explain the rise in adolescent suicidal behavior. Poor sleep is a potent risk factor for suicide and suicidal behavior, and prevalence of inadequate sleep duration in adolescents has increased over the past 15 years.^27,28^ The increase in exposure to social media among youths parallels the increase in adolescent suicidal behavior.^29^ However, the way that social media is used, rather the amount of exposure may be the source of its deleterious effects. Social media use that results in cyberbullying, interferes with sleep, is addictive, or involves social comparison rather than social connection is more likely than overall use to predict adverse mental health outcomes, including suicidal behavior.^29,30^

Can we use this information to reverse the increase in adolescent suicide? Improvements in access and quality of treatment for parents with ARD and SUD could help to reduce mortality and morbidity due to parental DoD.^31^ Interventions for families affected by DoD that include children can help to attenuate suicidal risk in youths, especially since interventions for parents with SUD are more likely to be successful if they include parenting components.^32^ Psychopathology, sleep problems, and chronic pain in adolescence predispose adolescents to the development of DoD in adulthood.^33,34,35^ There are effective universal school- and primary care–based interventions that target youths and their families to prevent the development of substance use and suicidal behavior.^36,37^ The Fast Track intervention, an intensive intervention for youths at risk for conduct disorder, showed efficacy in preventing the development of adulthood DoD.^38^ Better management of physical and mental health conditions, including sleep problems, in adolescence that predispose to the development of DoD may also prevent the onset of DoD in adulthood.^34,35,39,40^ Collaborative care models for both parents and youths could also improve case-finding, access to care, and outcomes.^41,42^ Finally, interventions like the Family Bereavement Program can substantially reduce the rates of suicidal ideation and behavior among youth who have lost a parent.^43^

### Strengths and Limitations

The main strengths of this study are the large size of the claims database, the ability to associate parental psychopathology with child outcomes, and the demonstration that these findings persisted even after propensity score weighting of family characteristics and adjustment for child age, sex, and psychopathology. Using similar methods, we previously showed an association of parental opiate misuse with offspring suicidal events.^15^ However, to our knowledge, this is the first direct demonstration of an association of parental DoD with adolescent suicidal behavior.

This study also has limitations. This database consisted of individuals with private insurance, which is not representative of the US population. Deaths of despair were initially reported to be most common in poorer, less well-educated individuals, so that one might expect that the rates of these disorders to be higher in individuals with Medicaid or no insurance. Moreover, as with every insurance claims database, we could only report on those who received treatment, and not on all affected individuals. Claims data are based on clinical assessment and coding, which can be subject to error. Race and ethnicity data were not available, limiting our ability to examine health disparities. There was no way to establish whether parents and children were biologically related. While there are many adverse family experiences associated with parental DoD, this dataset did not include them. Additionally, we showed an association of parental DoD with adolescent suicidal behavior, rather than the much rarer outcome of adolescent suicide. However, adolescent SAs are the single most potent risk factor for adolescent suicide,^44,45^ and other studies have shown an association of each of the DoDs, SAs, and suicide death in offspring, including adolescents.^16,23^

## Conclusions

In this cohort study of parents with and without DoD, we found that exposure to parental DoD was associated with a 2-fold increased risk for offspring SE, supporting a potential association of increases in parental DoD with youth suicidal behavior. Improved access to evidence-based care can reduce the morbidity and mortality in adults affected by DoD. Family-based interventions for these families could identify youths at risk and repair disrupted family processes to prevent the deleterious sequelae that exposure to parental DoD confers on their offspring. Finally, the deployment of evidence-based preventive interventions to reduce youth antisocial behavior and SUD could potentially help to prevent the next generation of parents from being prey to these despairing conditions.

## Notes

Supplement 1. **eTable 1.** *ICD-9*/*ICD-10* Codes for Diseases of Despair **eTable 2. **Unweighted and Weighted ORs (95% CIs) From Genedralized Estimating Equation Models **eTable 3.** Unweighted and Weighted Rate per 10 000 Person-Year and HRs (95% CIs) **eTable 4.** Weighted Rate per 10 000 Person-Year and HRs (95% CIs) by Sex and Age

Supplement 2.
Data Sharing Statement
