# Supplementary material for: Parental Diseases of Despair and Suicidal Events in Their Children
Source: JAMA Netw Open. 2025 Sep 12;8(9):e2531442. doi: 10.1001/jamanetworkopen.2025.31442 (PMC12432636; doi:10.1001/jamanetworkopen.2025.31442)
Supplement: Supplement 1. — eTable 1. ICD-9/ICD-10 Codes for Diseases of Despair eTable 2. Unweighted and Weighted ORs (95% CIs) From Genedralized Estimating Equation Models eTable 3. Unweighted and Weighted Rate per 10 000 Person-Year and HRs (95% CIs) eTable 4. Weighted Rate per 10 000 Person-Year and HRs (95% CIs) by Sex and Age [file jamanetwopen-e2531442-s001.pdf]

# Supplemental Online Content

Brent DA, Hur K, Gibbons JB, Porta G, Gibbons RD. Parental diseases of despair and suicidal events in their children. *JAMA Netw. Open*. 2025;8(9):e2531442. doi:10.1001/jamanetworkopen.2025.31442

**eTable 1.** *ICD-9/ICD-10* Codes for Diseases of Despair

**eTable 2.** Unweighted and Weighted ORs (95% CIs) from Generalized Estimating Equation Models

**eTable 3.** Unweighted and Weighted Rate per 10 000 Person-Year and HRs (95% CIs)

**eTable 4.** Weighted Rate per 10 000 Person-Year and HRs (95% CIs) by Sex and Age

This supplemental material has been provided by the authors to give readers additional information about their work.

© 2025 Brent DA et al. *JAMA Network Open*.

eTable 1: ICD-9/ICD-10 Codes for Diseases of Despair

| Category | ICD-9 or ICD-10 Codes |
| --- | --- |
| Suicide Attempt and Intentional Self-Harm | ICD-9: E950-E959<br>ICD-10: X71-X83, T1491,<br>T36–T50 with the 6th character of the code = 2<br>(except for T36.9, T37.9, T39.9, T41.4, T42.7, T43.9, T45.9, T47.9, and T49.9, which are included if the 5th character of the code = 2)<br>T51-T65 with the 6th character of the code = 2<br>(except for T51.9, T52.9, T53.9, T54.9, T56.9, T57.9, T58.0, T58.1, T58.9, T59.9, T60.9, T61.0, T61.1, T61.9, T62.9, T63.9, T64.0, T64.8, and T65.9, which are included if the 5th character of the code = 2)<br>T71 with the 6th character of the code = 2 |
| Drug-Related Diseases | ICD-9: 304, 850–858, 962, 980–980.5<br>ICD-10: F11–F16, F19, X40–X45, X85, Y10–Y15 |
| Alcohol-Related Diseases | ICD-9: 291, 303, 571<br>ICD-10: F10, K70, K73–K74 |

© 2025 Brent DA et al. *JAMA Network Open*.

eTable 2: Unweighted and Weighted ORs (95% CIs) from Generalized Estimating Equation Models

| Category | Adjustment | Clustering | OR (95% CI) |
|---|---|---|---|
| Unweighted | No | No | 2.26 (2.08 - 2.45) |
| Weighted | No | No | 1.82 (1.67 - 1.97) |
| Unweighted | No | Yes | 2.26 (2.08 - 2.45) |
| Weighted | No | Yes | 1.82 (1.64 - 2.01) |
| Unweighted | Age + Sex | No | 2.10 (1.94 - 2.28) |
| Weighted | Age + Sex | No | 1.67 (1.54 - 1.82) |
| Unweighted | Age + Sex | Yes | 2.10 (1.93 - 2.28) |
| Weighted | Age + Sex | Yes | 1.67 (1.51 - 1.85) |
| Unweighted | Age + Sex + Disease Conditions | No | 1.68 (1.55 - 1.83) |
| Weighted | Age + Sex + Disease Conditions | No | 1.48 (1.36 - 1.61) |
| Unweighted | Age + Sex + Disease Conditions | Yes | 1.68 (1.54 - 1.83) |
| Weighted | Age + Sex + Disease Conditions | Yes | 1.48 (1.34 - 1.64) |

eTable 3: Unweighted and Weighted Rate per 10,000 Person-Year and HRs (95% CIs)

| Age Group | Category | # of Adults | Group | # of Kids | # of Events | # of Days | Rate per 10K PY | HR (95% CI)[1] | HR (95% CI)[2] | HR (95% CI)[3] |
|---|---|---|---|---|---|---|---|---|---|---|
| All Ages | Unweighted | One | Non-DoD | 755,747 | 563 | 320,454,736 | 6.41 | | | |
| | | | DoD | 751,510 | 1,084 | 363,414,425 | 10.89 | 1.67 (1.51-1.85) | 1.69 (1.52-1.87) | 1.36 (1.23-1.51) |
| | Unweighted | Both | Non-DoD | 988,435 | 582 | 530,409,555 | 4.01 | | | |
| | | | DoD | 65,623 | 126 | 34,888,466 | 13.18 | 3.29 (2.72-3.99) | 2.86 (2.36-3.47) | 2.12 (1.75-2.58) |
| | Weighted | One | Non-DoD | 550,223 | 445 | 233,926,004 | 6.95 | | | |
| | | | DoD | 1,133,107 | 1,384 | 546,059,027 | 9.25 | 1.31 (1.18-1.46) | 1.31 (1.18-1.46) | 1.18 (1.06-1.31) |
| | Weighted | Both | Non-DoD | 716,532 | 442 | 385,145,404 | 4.19 | | | |
| | | | DoD | 96,364 | 172 | 51,122,594 | 12.26 | 2.93 (2.46-3.49) | 2.54 (2.13-3.03) | 2.06 (1.72-2.46) |

[1] Hazard ratio without adjustment for age and sex

[2] Hazard ratio with adjustment for age and sex

[3] Hazard ratio with adjustment for age, sex, and baseline disease conditions

© 2025 Brent DA et al. *JAMA Network Open*.

eTable 4: Weighted Rate per 10,000 Person-Year and HRs (95% CIs) by Sex and Age

| Gender | Age Group | Group | # of Youths | # of Events | # of Days | Rate per 10K PY | HR (95% CI)[1] | HR (95% CI)[2] | HR (95% CI)[3] |
|---|---|---|---|---|---|---|---|---|---|
| Male | All Ages | Non-DoD | 646,546 | 222 | 316,020,796 | 2.57 | | | |
| | | DoD | 627,808 | 349 | 304,666,702 | 4.18 | 1.63 (1.38-1.93) | 1.51 (1.28-1.79) | 1.35 (1.14-1.60) |
| | Ages 8-11 | Non-DoD | 328,444 | 42 | 159,950,583 | 0.95 | | | |
| | | DoD | 292,801 | 36 | 141,850,950 | 0.94 | 0.99 (0.63-1.54) | 0.97 (0.62-1.51) | 0.85 (0.54-1.33) |
| | Ages 12-15 | Non-DoD | 318,102 | 180 | 156,070,212 | 4.22 | | | |
| | | DoD | 335,007 | 313 | 162,815,753 | 7.01 | 1.66 (1.39-2.00) | 1.63 (1.36-1.96) | 1.46 (1.21-1.75) |
| Female | All Ages | Non-DoD | 620,209 | 666 | 303,050,612 | 8.02 | | | |
| | | DoD | 601,663 | 1,206 | 292,514,919 | 15.05 | 1.88 (1.71-2.07) | 1.73 (1.57-1.90) | 1.53 (1.39-1.68) |
| | Ages 8-11 | Non-DoD | 315,686 | 30 | 153,793,604 | 0.71 | | | |
| | | DoD | 281,376 | 82 | 136,510,623 | 2.20 | 3.12 (2.05-4.74) | 2.99 (1.97-4.55) | 2.80 (1.84-4.26) |
| | Ages 12-15 | Non-DoD | 304,523 | 636 | 149,257,009 | 15.54 | | | |
| | | DoD | 320,287 | 1,123 | 156,004,296 | 26.29 | 1.69 (1.54-1.87) | 1.67 (1.52-1.84) | 1.47 (1.33-1.62) |

[1] Hazard ratio without adjustment for age and sex

[2] Hazard ratio with adjustment for age and sex

[3] Hazard ratio with adjustment for age, sex, and baseline disease conditions

© 2025 Brent DA et al. *JAMA Network Open*.
