# Supplementary material for: Parental Diseases of Despair and Suicidal Events in Their Children
Source: JAMA Netw Open. 2025 Sep 12;8(9):e2531442. doi: 10.1001/jamanetworkopen.2025.31442 (PMC12432636; doi:10.1001/jamanetworkopen.2025.31442)
Supplement: Supplement 2. — Data Sharing Statement [file jamanetwopen-e2531442-s002.pdf]

Data Sharing Statement

Brent. Parental Diseases of Despair and Suicidal Events in Their Children. *JAMA Netw Open*. Published September 12, 2025. doi:10.1001/jamanetworkopen.2025.31442

Data
Data available: No

Additional Information
Explanation for why data not available: All data used in this study were obtained from Merative (formerly IBM Watson Health) as a part of their MarketScan database under license to the University of Chicago.
